# Tracking priming-induced language recovery in aphasia with pre-trained language models

**DOI:** 10.3389/frai.2025.1668399

**Published:** 2025-10-30

**Authors:** Yan Cong, Jiyeon Lee

**Affiliations:** ^1^School of Languages and Cultures, Purdue University, West Lafayette, IN, United States; ^2^Center on Aging and the Life Course, Purdue University, West Lafayette, IN, United States; ^3^Department of Speech, Language and Hearing Sciences, Purdue University, West Lafayette, IN, United States

**Keywords:** GenAI, large language models, language rehabilitation, aphasia recovery, structural priming as treatment, prompt engineering, aphasia, automatic clinical assessment

## Abstract

This study explores the use of pre-trained language models (PLMs) in tracking priming treatment induced language recovery in aphasia. We evaluate PLM-derived surprisals, the negative log-probabilities of a word or a sequence of words calculated by a PLM given its preceding context, as a continuous and interpretable measure of treatment-induced language change. We found that surprisal scores decreased following structural priming treatment, especially in participants with more severe sentence production impairments. We also introduce a prompting-based pipeline for clinical classification tasks. It achieved promising results in classifying aphasia sentence correctness (F1 = 0.967) and detecting error categories in aphasia (accuracy = 0.846). Such use of PLMs for modeling, tracking, and automatically classifying language recovery in aphasia represents a promising deployment of GenAI in a clinical rehabilitation setting. Together, our PLM-based analyses offer a practical approach for modeling language rehabilitation, tracking not only language structure but also individual change over time in clinical contexts.

**Clinical trial registration:**

Identifier NTC05415501.

## 1 Introduction

Computational methods involving pre-trained language models (PLMs) have recently shown promise in adapting models trained on typical speech to better accommodate atypical speech patterns (e.g., Purohit et al., 2023; Müller-Eberstein et al., 2024; Cong et al., 2024a; García et al., 2024). For individuals with atypical speech, such as people with aphasia (PWA), an acquired language disorder caused by brain injuries, language recovery is a challenge. Although a range of treatments has been developed, substantial individual variability in treatment outcomes continues to impede the development of reliable predictive models for recovery. Recently, AI has been increasingly used for aphasia intervention (e.g., Bailey et al., 2024; Imaezue and Marampelly, 2025). More relevant to the current study, previous AI-related research has demonstrated that PLM-derived scores such as PLM-surprisals (the negative log-probability of an utterance or a word given its preceding context as computed by a PLM) seem effective in detecting the presence and types of aphasia during discourse tasks (Rezaii et al., 2023; Cong et al., 2024a,b). Building on this line of work, the current study investigates whether PLMs can also serve as a sensitive utility to track language changes in aphasia following a language training, specifically structural priming training.

Structural priming, the unconscious repetition of previously encountered syntactic structures across otherwise unrelated sentences, plays a critical role in language processing and learning (Bock and Griffin, 2000; Pickering and Ferreira, 2008; Branigan and Pickering, 2017). Growing evidence has documented therapeutic potential of structural priming in improving language production and comprehension in PWA by facilitating access and use of syntactic representations (Lee, 2024, for review). Prior studies have demonstrated that PWA benefit from implicit structural priming, as evidenced by lasting improvements in syntactic production (Hartsuiker and Kolk, 1998; Lee et al., 2019b, 2024; Lee and Man, 2017; Man et al., 2019) and comprehension (e.g., Keen and Lee, 2022; Lee et al., 2019a). The present study leverages conditional surprisals (the negative log-probabilities of a participant’s production conditioned on a target production) derived from PLMs as a metric, and uses prompt engineering of PLMs to automatically assess priming-induced language learning in aphasia. By assessing the divergence between participants’ productions and the target sentence, our methodology quantifies the degree to which structural priming training brings aphasia closer to expected linguistic outputs. Concretely, we examined both group-level training effects and individual variability in response to intervention.

In Study 1, we tested whether PWA and age- and education-matched control participants (AEM) would show significant reductions in PLM-surprisals following structural priming. We also asked if priming-induced changes in surprisals measures would be greater for PWA than controls and reflect individual differences within the PWA group. Study 2 further explored the relationship between surprisals measures and specific production error types, thereby spelling out how these metrics reflect not only local lexical recovery but also broader syntactic restoration. Study 3 used few-shot learning to prompt PLMs, a technique used to enhance the performance of PLMs by presenting a limited number of examples (or “shots”) within the prompt. These examples serve as demonstrations of the desired output format task. Specifically in the current study, we used this few-shot prompting technique for automatic assessment and tracking of language recovery. This is achieved by measuring the alignment between participants’ productions and target sentences, and by classifying error types when misalignment occurs. This approach addresses the question of automation and enhances the efficiency of language rehabilitation assessment.

Our findings suggested the feasibility, efficacy, reliability, and interpretability of PLMs as clinical utilities, paving the way for more targeted and effective interventions in language recovery and therapy. First, our results showed that PLMs offer a promising avenue for detecting subtle changes in priming-induced language production. Aphasia recovery is highly variable, some individuals show substantial gains, while others progress slowly, highlighting the need for more precise and personalized tools to predict treatment responsiveness over time. Classic clinical measures often fail to capture the nuanced linguistic patterns that underlie this variability. We provided results that PLM-derived surprisals, which integrate syntactic as well as lexical information into a single metric, can serve as an interpretable and individualized marker of priming-induced language recovery. Further, our results indicated that prompting PLMs enables automated assessment of priming-induced recovery, which not only streamlines the assessment but also opens the door to scalable, data-driven methods for monitoring language-related rehabilitation.

## 2 Related work

### 2.1 Structural priming in language learning and recovery

Structural priming is a key phenomenon in studies of language processing and learning. It refers to the unconscious repetition of previously encountered sentence structures during subsequent production and comprehension (Bock, 1986; Pickering and Ferreira, 2008). For example, if a language user hears a double object (DO) dative prime sentence (e.g., *the man is giving the woman flowers*), they are more likely to describe a new dative event in the double-object form (e.g., *the singer is giving the boy a guitar*), compared to when they heard a prepositional object (PO) prime sentence (e.g., *the man is giving flowers to the woman*). Importantly such priming effects last over intervening fillers and across sessions in both young children and adults (Branigan and McLean, 2016; Savage et al., 2006; Heyselaar and Segaert, 2022). Thus, structural priming is thought to reflect how speakers implicitly learn to map messages onto certain syntactic structures through prior linguistic experiences (Bock and Griffin, 2000; Chang et al., 2012).

Implicit learning theories provide a framework for understanding how structural priming functions in language acquisition and learning. In a commonly cited theory, Chang and colleagues characterized structural priming as a consequence of prediction-error based language learning (Chang et al., 2006, 2012; but see Pickering and Ferreira, 2008 for additional accounts of structural priming). They proposed that as the speaker processes a prime sentence incrementally, they make predictions about upcoming word order. If the experienced (primed) linguistic input (e.g., DO dative sentence) is different from what they expected (e.g., PO dative) structure, this discrepancy drives adjustments in their syntactic processing system, biasing them to produce primed structures more frequently over time. In fact, many studies report structural priming results in enduring changes in various populations, including young children (Rowland et al., 2012; Branigan and McLean, 2016), second language learners (Shin and Christianson, 2012), and in both children and adults with language disorders (Leonard, 2011; Lee, 2024, for review).

In aphasia, where language production is often impaired, using syntactic repetition as a general strategy to simulate use of more complex and fluency speech has a long history, although its long-term benefits remain equivocal (e.g., Helm-Estabrooks, 1981; Doyle et al., 1987; Fink et al., 1995). For example, Sentence Production Program for Aphasia (formerly HELM Elicited Language Program for Syntax Stimulation or HELPSS; Helm-Estabrooks, 1981) emphasizes repeated practice of various syntactic structures in story contexts to remediate agrammatic speech. More recently, structural priming has been recognized for its potential to create lasting changes in sentence production in PWA. Growing findings suggest that it facilitates language (re-)learning in aphasia, rather than simply boosting immediate access of primed structures (e.g., Cho-Reyes et al., 2016; Lee et al., 2024; Lee and Man, 2017; Keen and Lee, 2022; van Boxtel et al., 2023; Lee et al., 2024; Rainey et al., (in press)). PWA demonstrate significant priming effects that persist over up to 10 intervening filler utterances between a prime and a target sentence (e.g., Cho-Reyes et al., 2016; Man et al., 2019; Lee et al., 2019a, 2019b). PWA show cumulative improvements in trained and untrained stimuli over repeated priming sessions or trials, with cumulatively increasing effects over sessions (Lee and Man, 2017; Rainey et al., (in press); Lee et al., 2024; van Boxtel et al., 2023; Zhang and Lee, 2025). Notably, while some studies primarily recruited participants with agrammatic Broca’s aphasia (Cho-Reyes et al., 2016; Hartsuiker and Kolk, 1998), others recruited participants with varying aphasia types and found positive priming effects across aphasia types (e.g., Yan et al., 2018; Keen and Lee, 2022; Lee et al., 2024; Saffran et al., 1988).

Another line of evidence that structural priming facilitates language learning comes from the so-called *inverse preference effect*, where individuals with less proficient language skills tend to show larger priming effects. Branigan and Messenger (2016), for example, found that children, as less proficient language users, exhibited stronger syntactic priming effects than adults, especially for long-term priming conditions. Hartsuiker and Kolk (1998) found priming effects only in PWA, examining production of passive and DO dative constructions, but not in controls. van Boxtel et al. (2023) found that PWA exhibited greater cumulative adaptation in sentence planning processes, as measured by eye tracking, to priming compared to controls. Additionally, Cho-Reyes et al. (2016), although failed to find increased priming effects for PWA at the group level, they found that within the PWA group, individuals with more severe sentence production impairments showed larger priming effects. However, the inverse preference effect was not supported in all studies. Yan et al. (2018) have found comparable priming effects between PWA and controls and Man et al. (2019), for example, have found reduced priming effects in PWA compared to controls. Nonetheless, these findings highlight the variability in priming responses among individuals with aphasia and the possibility that those with greater sentence production difficulties would show greater priming-induced improvements.

### 2.2 Structural priming in PLMs

The study of structural priming has also become an area of interest within the field of natural language processing recently, specifically concerning PLMs. Recent investigations have suggested that PLMs exhibit structural priming effects similar to those observed in human language users. Pertaining to the current study, Jumelet et al. (2024) demonstrated that PLMs are susceptible to structural priming across various conditions. Their experiments revealed that PLMs generate higher rates of syntactic alignment when exposed to specific sentence structures, reflecting the structural tendencies exhibited by human speakers. This indicates that PLMs do not merely rely on rote memorization but might be capable of abstracting grammatical relationships from their training data, facilitating a process that seems to mirror human syntactic processing and reuse. Jumelet et al. (2024) also suggested that structural priming in PLMs is heavily influenced by the models’ exposure to diverse syntactic structures during training. This training incorporates a multitude of patterns from large text corpora, likely allowing PLMs to represent and later reproduce similar structures when prompted. Such behavior indicates the models’ tendency to lead to coherence and fluency in generated text, facilitating interactions that are more aligned with natural human dialogue.

More recently, Sinclair et al. (2022) explored structural priming in PLMs, finding that autoregressive models favor sentences structurally similar to their prefixes across various constructions, resembling structural priming seen in humans. Along with findings from van Schijndel and Linzen (2018), Prasad et al. (2019), and Sinclair et al. (2022) suggested that PLMs recognize structural similarities between sentences and anticipate repeated structures. While these studies do not focus on input length specifically, their contextual manipulations inherently involve length variations, leaving open the question of how structural properties interact with different input lengths.

Moreover, the intersection of structural priming with multilingual contexts has been examined. Previous studies indicated that multilingual PLMs exhibit cross-linguistic structural priming, where exposure to similar structures in one language can prime analogous structures in another (Michaelov et al., 2023). Michaelov et al. (2023) measured cross-lingual structural priming across eight experiments spanning six languages, plus four monolingual experiments in three non-English languages. Results revealed that models exhibit abstract grammatical representations similar to humans, influencing text generation across languages and demonstrating shared structural processing in multilingual models. The implications of structural priming for PLM performance and applications are multifaceted (Sinha et al., 2022). For instance, harnessing structural priming could enhance dialogue systems and conversational agents, allowing for more human-like interactions. By incorporating mechanisms of structural priming, developers can create PLMs capable of generating contextually relevant and grammatically coherent responses, which may lead to improved user experiences in natural language applications (Cai et al., 2023).

Another line of work concerns whether structural priming in PLMs reflects implicit learning mechanisms, as in humans, or merely the reuse of patterns from training data. To address this, studies such as Jumelet et al. (2024) and Sinclair et al. (2022) used controlled prefixes that varied syntactic structure while holding lexical content constant. Their findings showed that exposure to specific constructions (e.g., double-object versus. Prepositional-dative frames) influenced subsequent outputs, even with novel lexical items, suggesting abstraction over syntax rather than simple memorization. Nevertheless, the mechanism in these PLMs differs from human priming. For humans, priming is often linked to shared abstract representations guiding production. In PLMs, structural priming may have been understood as an emergent property of distributional learning. The effect likely arises from probabilistic next-token prediction shaped by large-scale exposure to structural patterns (Van Schijndel and Linzen, 2018; Kassner and Schütze, 2019; Kassner et al., 2020; Jurafsky and Martin, 2025).

## 3 Methods

### 3.1 Human participants

The human data reported here are from a total of 40 participants, including 24 participants with post-stroke aphasia (PWA) and 16 age- and education-matched control (AEM) who participated in a larger clinical trial project that examines the efficacy of structural priming training in aphasia (Clinical Trial registration No: NTC05415501).

PWA and AEM were matched for age (PWA Mean (*M*) = 59.2, SD = 11.4; AEM Mean = 63.4, SD = 11.1; *t* = −0.988, *p* > 0.05) and years in education (PWA Mean = 16.3, SD = 1.79; AEM Mean = 16.7, SD = 1.35; *t* = −0.722, *p* > 0.05). All participants were native speakers of English, with no known history of neurological or psychiatric disorders that could affect communication (besides the stroke for PWA). AEM controls were screened for their cognitive-linguistic skills using the Cognitive-Linguistic Quick Test Plus (Helm-Estabrooks, 2017) prior to study participation. All of them scored within normal limits on the composite severity rating score (*M* = 3.92/4.0, SD = 0.14, range 3.6–4.0) that was calculated across the subdomains of attention, memory, executive function, language, and visuospatial skills.

All PWA were at least 6 months post onset of stroke at the time of study enrollment (Mean = 81.3, SD = 66.8, range = 23–245 months). PWA completed a set of clinical tests to determine eligibility following the inclusion criteria as detailed in Lee et al. (2024). The tests included Western Aphasia Battery—Revised (WAB-R; Kertesz, 2007), Northwestern Assessment of Verbs and Sentences (NAVS; Cho-Reyes and Thompson, 2012), portions of the Comprehensive Aphasia Test (CAT; Swinburn et al., 2004), the Philadelphia Comprehension Battery (PCB; Saffran et al., 1988), and the Spoken Word-Picture Matching subtest of the Psycholinguistic Assessment of Language Processing in Aphasia (PALPA; Kay et al., 1996).

PWA test scores are reported in Table 1. All participants presented with moderate-to-mild aphasia, as determined by the WAB-R aphasia quotient (AQ) 50 or higher to ensure that our tasks are doable for them. We included participants with varying aphasia types (4 Broca’s, 2 Transcortical Motor, 14 Anomic, 2 Conduction; 2 Wernicke’s), given that reduced sentence production, especially for those with non-canonical word order are found in both fluent and nonfluent aphasias (e.g., Man et al., 2019; McAllister et al., 2009; Cho-Reyes and Thompson, 2012; Yan et al., 2018). PWA had to demonstrate relatively intact comprehension of single words and yes/no questions (>80% on the PALPA spoken word-picture matching task, >80% on the CAT Comprehension of Written Words subtest; >5/10 WAB-R Auditory Comprehension subscore; >80% on the NAVS Verb Comprehension Test, and >40% on the NAVS Sentence Comprehension Test) (Thompson, 2012). However, PWA showed difficulty producing complex sentences as shown in the SPPT of the NAVS and a set of sentence probe tasks that were devised and administered as part of our clinical trial (see Lee et al., 2024). Additionally, the increasing word length and repeated trials subtests of the Apraxia Battery for Adults-Second Edition (ABA-2; Dabul, 2000) were administered and those who exhibited severe apraxia of speech were excluded. PWA passed informal screening for significant attention and memory deficits using the Symbol Cancellation and Design Memory subtests of the CLQT+ (Helm-Estabrooks, 2017). This study was reviewed and approved by Purdue University Institutional Review Board (IRB-2021-659).

**Table 1:** Language testing scores for PWA participants, with means (*μ*) and standard deviations (σ2) included.

ID	WAB-R					PALPA	NAVS					CAT	PCB
	AQ	Fluency	AC	Naming	Repetition	SWP %	VCT	VNT	ASPT	SPPT	SCT	Comp of written words (%)	Total (%)
1	93.6	9	10	8.8	10	93	100	81.8	87.5	93.3	93.3	87	97
2	73.6	6	6.4	8.8	7.6	98	100	95.5	96.9	76.7	93.3	80	78
3	92.8	9	10	9.0	9.4	98	100	77.3	100	96.7	100	100	100
4	82.7	6	9.9	8.1	9.4	98	95.5	81.8	96.9	93.3	100	87	100
5	91.7	9	10	8.9	10	100	100	81.8	100	96.7	100	93	98
6	65.3	4	8.5	6.5	7.7	98	100	40.9	56.3	16.7	96.7	87	88
7	81.4	6	8.9	8.6	8.2	93	100	59.1	84.4	56.7	66.7	100	88
8	78.1	5	9.6	7.8	8.7	98	100	50.0	87.5	76.7	96.7	87	93
9	78.9	6	9.7	7.9	6.9	98	100	81.8	87.5	76.7	86.7	87	90
10	77.7	6	7.9	9.2	6.8	93	100	100	93.8	60.0	73.3	93	88
11	63.5	8	6.5	4.4	5.9	80	81.8	50.0	81.3	3.3	66.7	80	63
12	64.5	5	8.0	5.1	6.2	85	90.9	54.6	84.4	16.7	70.0	73	80
13	75.7	4	9.0	7.8	9.1	98	95.5	63.6	93.8	86.7	93.3	93	88
14	92.0	9	9.8	9.4	8.8	98	100	90.9	100	90.0	93.3	87	95
15	93.2	9	9.9	8.8	9.9	100	100	90.9	100	90.0	100	80	95
16	76.3	6	8.0	7.8	8.4	85	90.9	63.6	78.1	16.7	66.7	73	70
17	72.0	5	7.8	7.9	7.3	93	100	72.7	87.5	40.0	70.0	87	77
18	74.6	6	7.4	8.8	7.1	93	90.9	72.7	78.1	63.3	90.0	93	72
19	85.6	6	10	9.1	8.7	98	100	95.5	96.9	96.7	93.3	100	95
20	69.9	4	8.5	8.9	5.6	100	100	81.8	81.3	3.3	53.3	100	72
21	71.4	6	7.6	8.5	5.6	93	95.5	86.4	90.6	10.0	43.3	100	75
22	87.1	6	9.8	9.2	9.6	95	100	100	93.8	96.7	100	93	100
23	79.2	6	9.8	7.5	7.3	98	100	86.4	93.8	60.0	93.3	87	93
24	78.9	6	8.3	8.1	9.1	95	95.5	45.5	87.5	70.0	76.7	80	78
*μ*	79.2	6.3	8.8	8.1	8.1	94.9	97.3	75.2	89.1	62.0	84.0	88.6	86.4
σ^2^	9.2	1.6	1.2	1.2	1.4	5.2	4.6	17.8	9.9	33.6	16.4	8.2	11.0

WAB-R, Western aphasia battery-revised; AQ, aphasia quotient; AC, auditory comprehension; PALPA, psycholinguistic assessments of language processing in aphasia; SWP, spoken word picture matching test; NAVS, Northwestern assessment of verbs and sentences; VCT, verb comprehension test; VNT, verb.

### 3.2 Structural priming training paradigm

**Figure 1:**
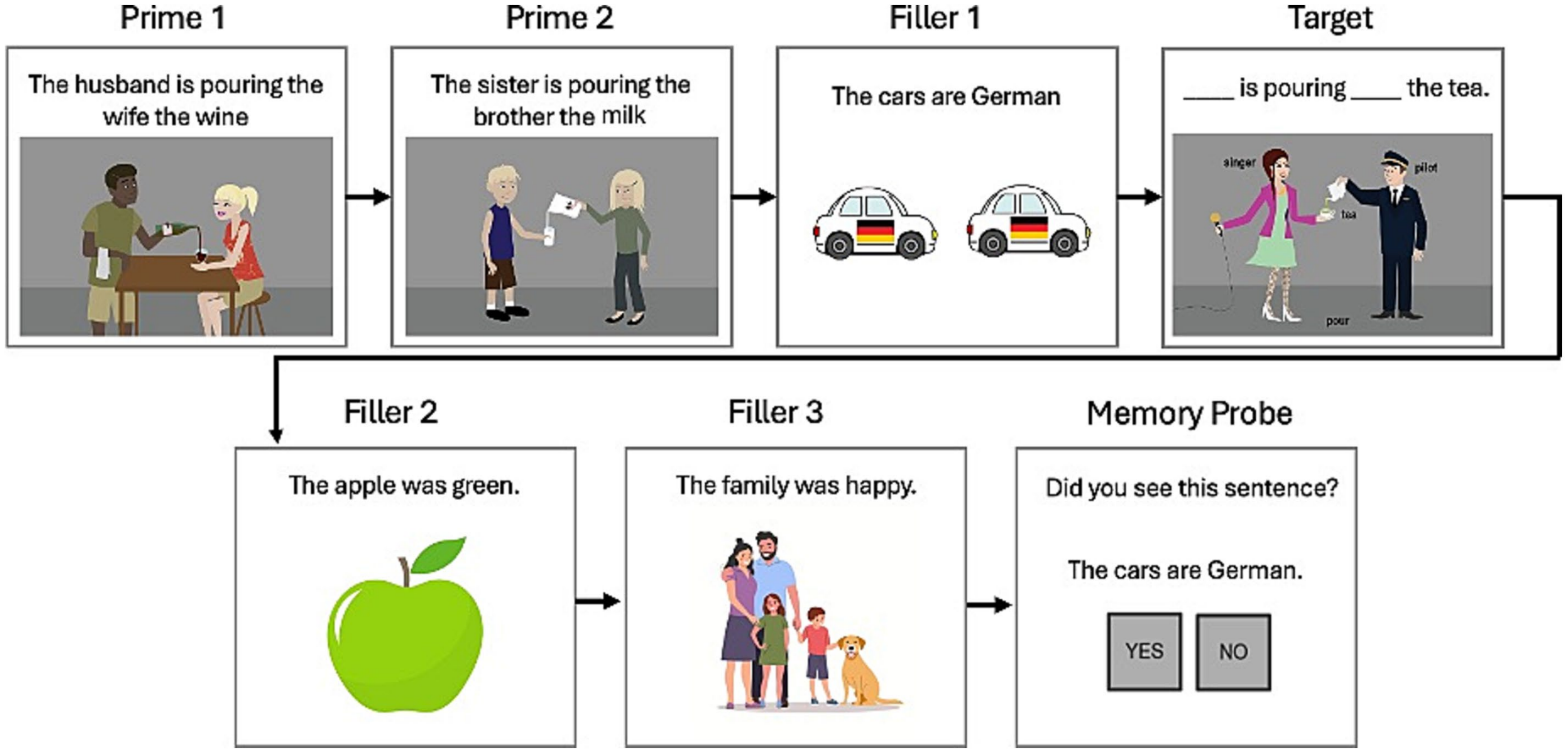
An example sequence of structural priming training trial for human participants.

Both groups of participants completed a structured priming training regimen, which consisted of Pre-test (before treatment), three sessions of structural priming training targeting production of DO dative sentences, and one-day (Post-test 1) and one-week post testing sessions (Post-test 2). The three training sessions were delivered over a two-week period, with at least 2 days between sessions. Each session consisted of 40 structural priming training trials as shown in Figure 1: participants first read two DO prime sentences (*Prime 1,2*) followed by a filler sentence (intransitive, as in *Filler 1*), then completed a target sentence (*Target*). After that, they read two additional filler sentences before finishing with a recognition probe. Each priming training session took about 30 min for AEM controls and 1–2 h for PWA. Training sessions were delivered either in-person or virtually over secure Zoom, depending on the participant’s availability for in-person sessions.

Before and one-day and one-week after the training, the participants completed a sentence production task, consisting of 15 trials involving dative alternations. We chose dative sentences as our stimuli, because they are among the most frequently studied and well-documented syntactic alternations in previous structural priming studies and English in both neurotypical speakers (Bock, 1986, 1990; Mahowald et al., 2016) and PWA (Lee, 2024, for review). Notably, the participants were presented with a unique set of 15 different stimuli at each testing time point to avoid practice effects. To prepare for the stimuli, a group of 15 high frequency (*M* = 3.985, SUBTLEXus corpus, Brysbaert and New, 2009), one-syllable dative verbs (e.g., *give, offer, show*) were selected and repeated with three different sets of nouns, yielding a total of 45 unique dative sentences (15/testing session). Across the three sets of stimuli that were administered at Pre-test, Post-test 1, and Post-test 2, we matched the frequency and lengths of the nouns (*M* number of syllables: 1.73, 1.6, and 1.64; M word frequency: 3.11, 2.97, and 3.12; all *p*’s > 0.05, independent *t*-tests). There was also no overlap between these stimuli used in the production task and the sentence stimuli used during the priming training sessions.

**Figure 2:**
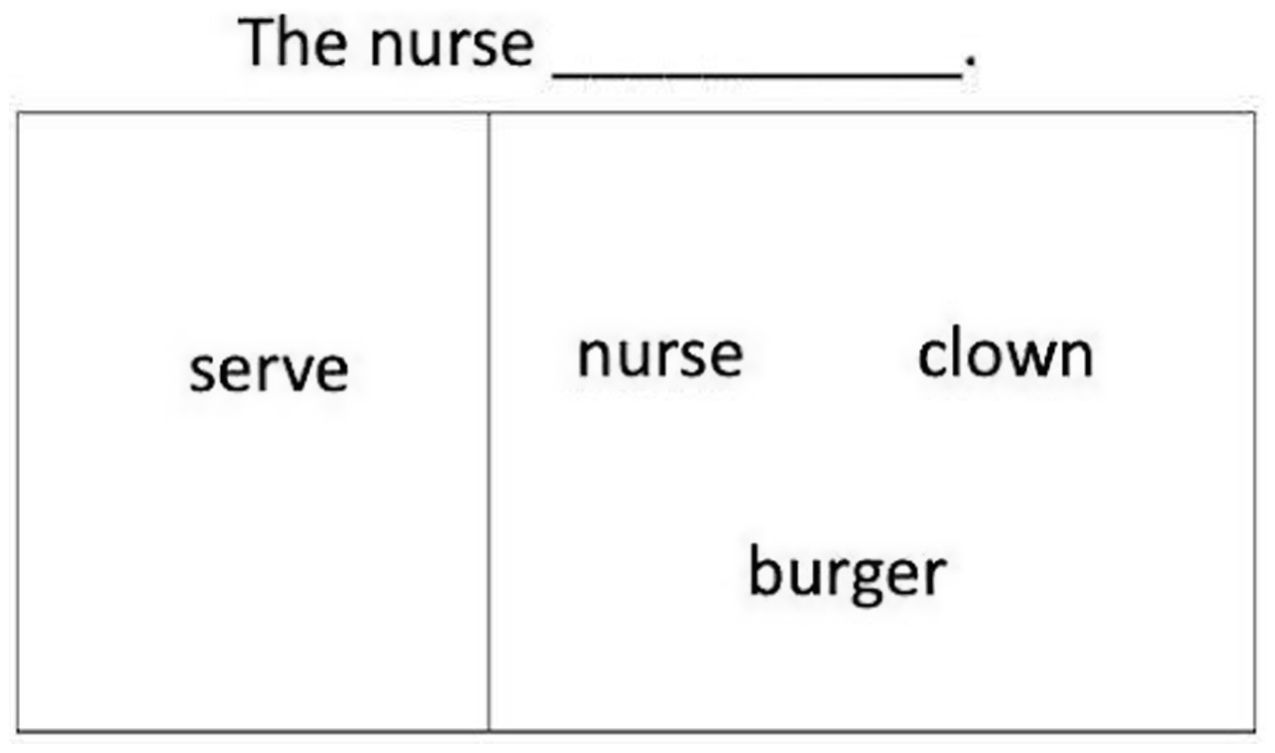
An example trial for the anagram production test.

As illustrated in Figure 2, in the sentence production task, participants saw an action verb like *serve* on the left of the screen and nouns like *nurse, clown, burger* on the right. A sentence frame like *The nurse ___* appeared at the top of the screen. Using all the given words and the frame, participants were asked to make a sentence. Participants were free to produce any type of sentences, including either DO or PO dative sentences. Because the priming training specifically aimed to improve preference of DO structures in the participants’ production responses, it was reasoned that if structural priming effectively changes participants’ syntactic production, our participants would be more likely to produce the DO, rather than the PO structure or any other alternative sentence types after training. Thus, our dependent measure of interest was whether the participants would show increased production of DO responses after priming training compared to before training.

### 3.3 Apply PLMs to track recovery

#### 3.3.1 Study 1. Do PLM-surprisals reveal training effects in sentence production?

Study 1 focused on training effects and the inverse preference phenomenon. We measured conditional surprisals across multiple time points (Pre-test, Post-test 1, and Post-test 2) to capture how structural priming training dative structures influences the alignment of participants’ sentence productions with expected targets, i.e., DO dative constructions. Each participant’s responses on the task were transcribed carefully by a group of trained researchers (*n* = 10). The data reported here is from a larger clinical trial study and were collected over the span of approximately 1 year. Each response’s correctness and error types were tallied for each participant. When scoring correctness of participants’ productions, we accepted synonyms that did not change the meaning of the sentence (e.g., *guy* for *man*; *ballerina* for *dancer*). However, we did not accept substitutions that changed the meaning of the sentence substantially (e.g., *professor* for *dancer*). Prior to serving as an independent coder, all research team members demonstrated 90% or higher inter-reliability in transcription and coding accuracies following the second author’s (JL) lab training protocol. All session data transcriptions and coding were double checked for any transcription errors by an independent coder who was not present in the testing session. Any confusions or disagreements were resolved through a group discussion with senior researchers of the study team, including the second author (JL). A blind inter-rater reliability was also established on randomly selected 20% of the data for accuracy and error coding every 3 months during data collection and the group was retrained if a consistent error was noted. Average inter-rater agreement rates were very high: 96.29% for accuracies (Cohen’s *κ* = 0.92) and 94.09% for error types (*κ* = 0.87).

The research team’s codes were used as the *ground truth labels*. In computational pipelines, a ground truth label is the correct, verified, and factual annotation for a piece of data (Jurafsky and Martin, 2025). These labels, determined through trained human researchers, serve as the benchmark or *true* answer for guiding and evaluating PLMs in our tasks. Prior to calculation of PLM-surprisals, disfluencies and annotation symbols, including parentheses, ellipses symbols, and paraphasia annotation symbols like *pp*, were removed from the transcribed data. After pre-processing, in total, the dataset consisted of 2,669 pairs of target sentence and participant production. There were 1,531 pairs in the PWA group and 1,138 in the AEM group. For the PWA group, there were 555 pairs annotated as “Correct,” and 976 as “Incorrect.” For AEM, there were 574 “Correct” and 564 “Incorrect.” Notably, “Incorrect” responses also include grammatical structures (e.g., PO dative, other sentence types) that are not the target DO structure, explaining for a relatively high number of “Incorrect” responses in AEM. In fact, when excluding non-target PO productions, the AEM group only had three incorrect responses for “Incorrect”; and for PWA, there were 231 for “Incorrect.”

We computed conditional surprisals using PLMs to capture how divergent the participants’ responses were from the expected DO target sentences. PLM-surprisals were derived from the conditional log-probability assigned by a PLM. We used *minicons* (Misra, 2022) default reduction function which averages the log-probability per token, providing a smoothed estimate of how probable a participant’s production is, given the target sentence. As a validated method in PLMs, NLP, and recent PLM-related aphasia research (Jumelet et al., 2024; Michaelov et al., 2023; Misra et al., 2020; Jurafsky and Martin, 2025; Rezaii et al., 2023), such smoothing enabled valid, aggregated comparisons of PLM-derived surprisals across different groups, time points, and PLMs. All surprisals analyses were conducted using *minicons*, with the exception of the individual trajectory regression. For this analysis, we implemented a custom pipeline using HuggingFace’s *Transformers* library (Wolf et al., 2019), allowing us to extract raw (unsmoothed) surprisals for each individual production. This approach provided a direct view of individual variation prior to smoothing. Mathematically, PLM-surprisals compute (Equation 1):


(1) $$\text{Surp}=-\sum_{i=1}^{n}\;\text{logP}\;(w_{i}|C,w_{1},\ldots ,w_{i-1})\;$$

In Equation 1, *P* is the participant production including *n* tokens (*w_1_, w_2_, w_n_*), and the context *C* is the expected target sentence. The conditional surprisal *Surp* is then calculated as the negative log-probability of token *w_i_* given *C* and previously generated *P* tokens. A higher surprisal value indicates that the PLM finds the participant production less predictable and more diverged given the target sentence. Consider an example, where *C* is the expected target sentence *The tailor lends the actor the umbrella* and *P* is the actual verbatim production *The tailor lended an umbreller to an actor*, *Surp* by GPT-2 is 5.45. In contrast, with the same *C* but a less aligned *P The tailor lended an book to an athlete*, *Surp* is 6.77. The divergence between the participant’s actual production and the target sentence is manifested in the increase of *Surp* from 5.45 to 6.77. Our main PLM was the autoregressive GPT-2 (Radford et al., 2019), since studies suggested that GPT-2 surprisals seem to provide better fit to human behavioral analyses (e.g., Shain et al., 2024). We additionally included llama-2-7b-hf (Touvron et al., 2023) and Mistral-7B-v0.1 (Jiang et al., 2023), in order to validate generalizability of the inverse preference effects across multiple PLMs. All PLMs in this paper are autoregressive, because their pretraining objective (next-word prediction) is inherently aligned with the concept of conditional surprisal (Jurafsky and Martin, 2025).

There were two primary hypotheses: one at the group level and one at the individual level. First, at the group level, we hypothesized that if the surprisals measure is sensitive to structural priming training gains, both groups AEM and PWA would show lower surprisals at Post-test 1 and Post-test 2 compared to Pre-test. Further, in line with the inverse preference effect, we hypothesized that PWA, compared to the AEM group, would show greater reduction in surprisals following the training, although the previous findings are somewhat mixed as discussed earlier. Also, at the individual level, we explored whether PWA with higher surprisals at Pre-test would show larger reduction in surprisals at Post-test 1 and Post-test 2 compared to those with lower Pre-test surprisals.

To approach these hypotheses, we conducted three analyses at the group level. First, we conducted the Wilcoxon signed-rank test (a non-parametric test that does not assume normal distribution) comparing PLM-surprisals across time points for both groups. Second, we constructed a linear regression model predicting slope difference across time points for both groups. We first extracted the slope from each individual regression model involving separate time points, then we calculated the slope differences for pairwise time points. Using these differences, we built a new linear regression model, with initial PLM-surprisals at Pre-test predicting the slope change. Third, we examined group differences at each time point using a linear mixed-effects model with least-squares means pairwise comparisons. The contrasts were computed as the difference in PLM-surprisal values between the AEM and PWA groups (AEM–PWA).

Further, two individual-level analyses were conducted. First, for each participant, a linear regression model was used to examine changes in surprisals across time, where the difference in surprisals between consecutive time points was calculated. Second, to examine whether reductions in PLM-surprisals were associated with improvements in accuracy at the individual level in the PWA group, for each participant, we calculated the change in PLM-derived surprisals and ground-truth accuracy (i.e., the “Correct” label) from Pre-test to Post-test 1, and from Post-test 1 to Post-test 2. Pearson correlations were then computed across participants using these individual change scores, linking surprisals reduction to accuracy gains. All statistical analyses were conducted in *R* (R Core Team, 2025).

#### 3.3.2 Study 2. What is PLM-surprisals tracking in language recovery?

Study 2 analyzed the interplay between various production categories and surprisals. Production error categories include errors which are grammatically incorrect and those that are grammatically correct but misaligned with the target sentence. We examined their relationships with surprisals over time. The purpose is to identify the specific production patterns associated with higher or lower surprisals and assess how these relationships evolve as PWA recover. We focused on PWA’s language recovery, and our analyses involved AEM controls in order to establish an interpretive baseline, benchmarking the comparison with our interested group - PWA. The same data pre-processing and PLM-surprisals pipeline used in Study 1 was applied in Study 2, to evaluate the overall deviation of the actual productions from the expected responses, taking into account various categories of non-target responses.

The evaluation process categorizes deviations from target sentence structures using predefined category codes, which are tallied by trained human annotators. Concretely, NT_po refers to grammatical non-target sentence structures, namely PO dative sentences such as *the man is giving the cake to the woman*, when the expected target sentence is *the man is giving the woman the cake*. Any other grammatical non-target structures are coded as NT_other (*the man and woman are enjoying cake*). Grammatical errors (GE) included argument structure violations errors, for instance, omitting obligatory arguments (*the man is giving*) or incorrect argument order (*the man is giving with the woman the cake*). The NS (non-sentential response) error category is when a response consists of a string of nouns (*man, woman, and cake*) or responses without lexical verbs. Lexical errors (LE) include incorrect verb or noun substitutions (*referee* was used for *king*) that deviate significantly from the correct target stimulus. Finally, other applies to a response with multiple error types or when none of the above categories applies.

There were two primary hypotheses. First, PLM-surprisals change across time points should be significantly associated with productions with errors, if surprisals can track language recovery as represented in distinct production error types. Second, we pinpointed that if PLM-surprisals capture local, lexical recovery, we would expect salient relationships between surprisals and lexical errors. On the other hand, if surprisals characterize beyond local, lexical recovery, we would predict significant relationships between surprisals and other grammatical categories, besides lexical errors.

To address these hypotheses, we conducted two analyses. First, we fit a linear mixed-effects model to examine whether PLM-derived surprisal values of error-containing productions predicted time point. Here, error productions refer to productions with error types, including GE, NS, and LE. Second, to further investigate the relationship between surprisals and production error types within the PWA group, we computed Spearman correlation coefficients and associated *p*-values between surprisals and the one-hot-encoded counts of each error category.

#### 3.3.3 Study 3. How effective is PLM-prompting in assessing language recovery?

Besides probability-based metrics such as PLM-surprisal, we evaluated the few-shot prompting approach. Few-shot prompting is a technique used to improve the performance of PLMs by presenting a limited number of examples (or “shots”) within the prompt (Jurafsky and Martin, 2025). These examples demonstrate a sample sentence and the desired label for correctness and error types. We used the ground truth annotations for correctness and error types, namely the benchmark or “true” answer for instructing and evaluating PLMs, as discussed in the previous Section 3.3.1. The examples were also from these ground truth annotations, validated by trained researchers, as discussed in Section 3.3.1. The same data pre-processing pipeline used in Study 1 and 2 was applied to Study 3.

A held-out sentence pairs set was used to construct demonstration examples for few-shot prompting. Each exemplar included a triplet of (1) the target sentence, (2) the pre-processed participant production, and (3) the binary correctness label (1 = correct, 0 = incorrect). Following Qiu et al. (2025), we employed a “one trial per run” procedure, meaning that each interaction session with the PLM involved only a single experimental trial: one pair of the target sentence and the production and the instruction prompt involving the triplet exemplar. This method can minimize biases from previous trials affecting the current judgment and help resolve an issue where PLMs occasionally lost track of instructions midway. Moreover, the shorter sessions inherent in this design were less susceptible to server or connectivity problems. For each test item, we assembled a prompt of the following form: *Evaluate the Participant Production against the Target Sentence by following the step-by-step instructions. Return 1 if the candidate uses a correct Double Object (DO) structure that matches the target’s meaning; otherwise return 0*. This excerpt was concatenated with stepwise instruction prompts.

This prompt template communicated the binary classification task and provided six in-context examples (three negative and three positive examples) to guide PLM’s classification and ground the PLM’s interpretation. We designed the prompts based on trained annotators’ judgments and validations and Jurafsky and Martin (2025), which suggested that too many examples might lead to overfitting, but too few might not provide PLMs with sufficient domain-specific information. We queried llama-3-8b-instruct (Touvron et al., 2023), deepseek-r1 (Guo et al., 2024), llama4-maverick (Meta AI, 2025), and gpt-4o (OpenAI, 2024), via a unified API: the Replicate API; API inquiry time from February to May 2025. Due to class imbalance, F1 scores were used as the primary metric rather than accuracy. We chose these PLMs, because they were open sourced, and they were benchmarked as some of the best PLMs in generation tasks at that moment (Jurafsky and Martin, 2025; https://huggingface.co/models).

We additionally prompted PLMs to assign an error type label by extending the instructions. We added examples to illustrate error-type detections. We modified the prompt template for “correctness” by breaking down error types into stepwise guidance. For the PWA group, our dataset included 745 samples tagged with NT_po, 152 with GE, 28 with Other, 25 with LE, 19 with NS; the AEM dataset included 561 samples tagged with NT_po, 1 with LE, 1 with NT_other, and 1 with Other. The same API-based prompting pipeline used in the binary classification task of “correctness” was applied to classify error types in productions marked as “0” (incorrect) in the original dataset, as verified by trained clinicians. This classification task was evaluated by the matching accuracy between PLM’s predicted and the ground-truth error-type labels.

For both prompting tasks (correctness and error types), prompt templates, all the examples, instructions, and all the step-wise details are available in the Open Science Forum at doi: 10.17605/OSF.IO/FMNSY.

## 4 Results

### 4.1 PLMs reveal training effects and inverse preference

Our results suggested that PLM-surprisals are informative of language recovery, particularly language learning in the PWA group. PLM-surprisals measures generally revealed that participants’ productions became more aligned with the expected DO dative structures over time following structural priming training. Our findings indicated such training effects at both the group and the individual level.

#### 4.1.1 Group level analyses

**Figure 3:**
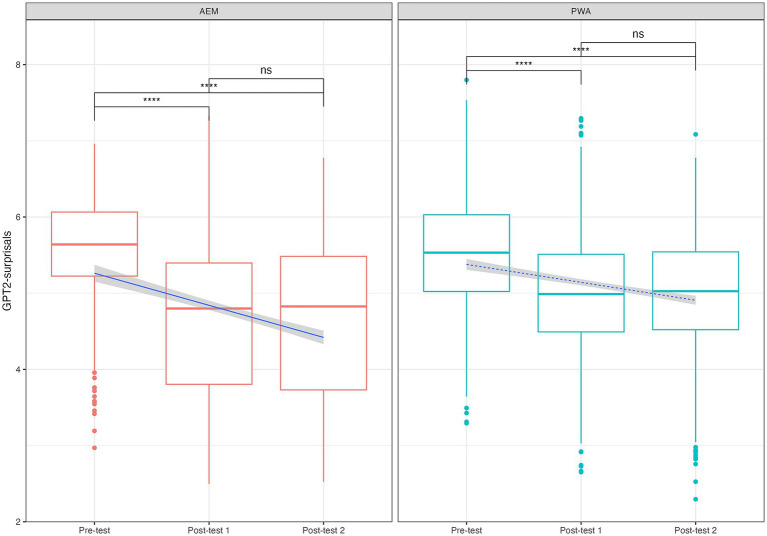
Surprisals time points comparisons, Bonferroni correction applied. ****p < 0.0001, ns: *p* > 0.05.

Figure 3 indicated that for both groups, there were significant decreases of surprisals from Pre-test to Post-test 1 and from Pre-test to Post-test 2, but not from Post-test 1 to Post-test 2. These suggested that participants’ sentence productions generally became aligned with target sentences following the training and the improvements were maintained over time.

Table 2 reinforced this group-level training effects by demonstrating that initial surprisals can predict the magnitude of change in subsequent training sessions such as Post-test 1 and Post-test 2. The slope differences between Pre-test and Post-test 1, and between Pre-test and Post-test 2, were significant for PWA (coefficients of 0.74 and 0.686 respectively, with relatively high adjusted *R*^2^ values 0.527 and 0.446). However, the difference between Post-test 1 and Post-test 2 was not significant (adjusted *R*^2^ 0.062). These results suggested that after training, PWA showed significantly reduced surprisals compared to Pre-test and the training effects were maintained at Post-test 2. Changes in AEM were also significant but less pronounced (coefficient of 0.673 and 0.689, respectively). The inverse preference effect was also manifested in surprisals. Both PWA and AEM groups showed significance and positive coefficient when using Pre-test surprisals predicting slope difference, with the PWA group slope change from Pre-test to Post-test 1 the most salient. The decrease of slope change from Pre-test to Post-test 1 was the largest (coefficient = 0.74), among all the coefficients. This indicated that the PWA group, with more impaired production in the beginning, showed larger amounts of improvements after structural priming training.

**Table 2:** Slope differences across time.

Group	𝛽	*SE*	*p*	Adjusted *R*^2^
Slope difference (PWA)				
Δ(Pre-test−Post-test 1)	0.740	0.144	3.63e-05	0.527
Δ(Pre-test−Post-test 2)	0.686	0.155	0.0002	0.446
Δ(Post-test 1−Post-test 2)	−0.321	0.202	0.126	0.062
Slope difference (AEM)				
Δ(Pre-test−Post-test 1)	0.673	0.198	0.004	0.414
Δ(Pre-test−Post-test 2)	0.698	0.199	0.004	0.447
Δ(Post-test 1−Post-test 2)	0.109	0.276	0.700	−0.064

Coefficients 𝛽 in Table 3 consistently showed significant differences between the AEM and PWA groups. The negative coefficient values suggested generally higher surprisals in the PWA than in the AEM groups. Further, the regression coefficients for Pre-test, Post-test 1, and Post-test 2 indicated statistically significant improvements, suggesting that increased exposure through structural priming training leads to more predictable (hence lower surprisals), target-like productions. The coefficients (absolute values) decrease from Pre-test to Post-test 1 and from Pre-test to Post-test 2, implying that the PWA group is catching up as their responses become more structured.

**Table 3:** Surprisals group comparison across time.

Time point	𝛽	SE	*p*
Pre-test	−0.901	0.170	<0.0001
Post-test 1	−0.608	0.166	0.008
Post-test 2	−0.653	0.166	0.004

#### 4.1.2 Individual level analyses

**Figure 4:**
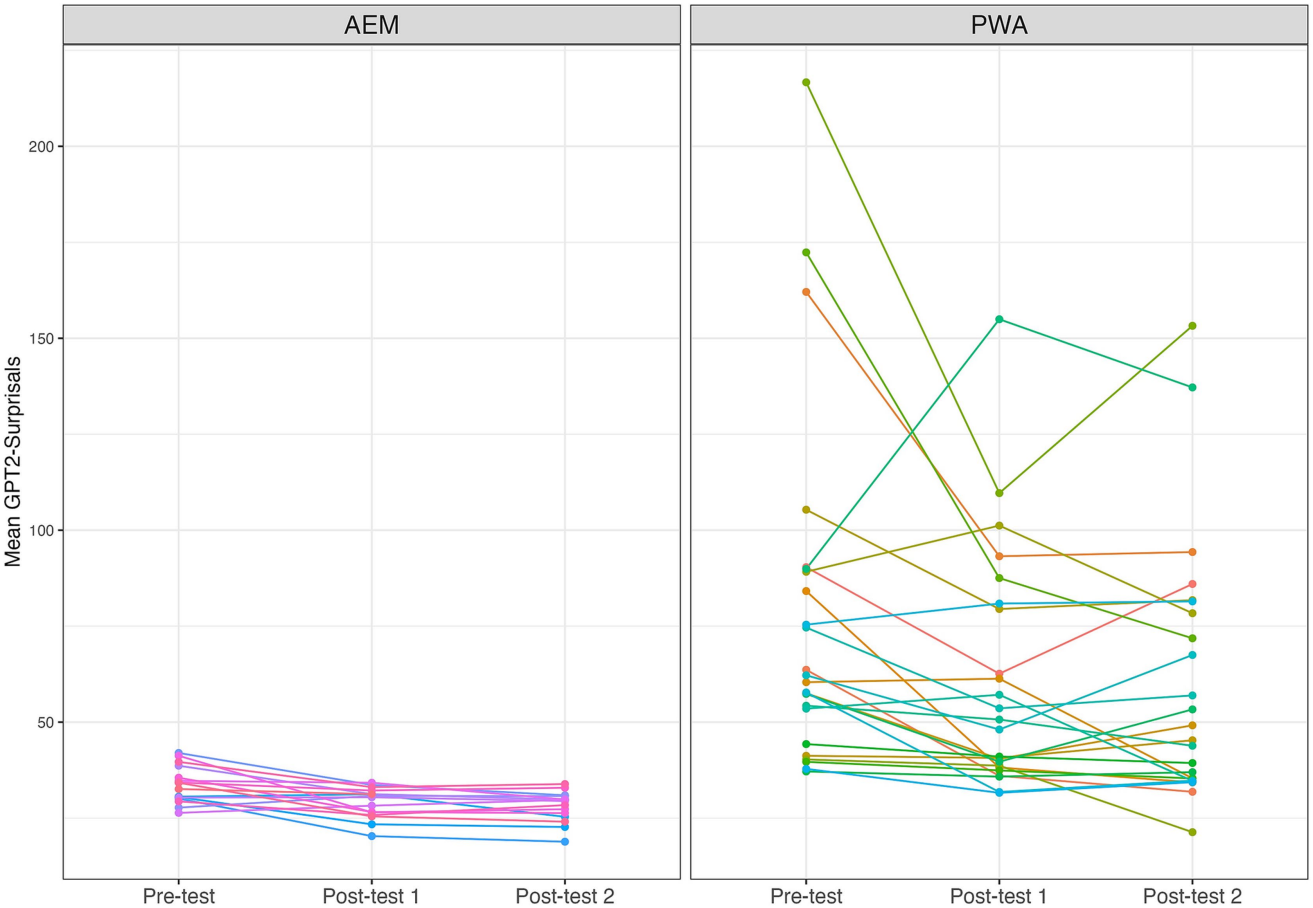
Trajectories of surprisals for each participant.

Figure 4 showed that these individual trajectories reveal a general pattern of decreasing surprisal values from Pre-test to Post-test 1, with this reduction often sustained at Post-test 2. This pattern was observed across both the AEM and PWA groups. Notably, individuals who began with higher surprisals at Pre-test tended to show greater reductions following training, suggesting a potential for surprisals to reflect the degree of individual responsiveness to intervention. Additionally, changes between Post-test 1 and Post-test 2 were minimal or nonsignificant for most participants, indicating a maintenance of training effects over time. To statistically justify the observable patterns in Figure 4, follow-up pairwise comparisons were conducted using the least-squares means pairwise analyses, the *emmeans* package in *R* (Lenth, 2024), based on a linear regression model with surprisals as the dependent variable predicting time points. Our result suggested that a total of 11 out of 24 PWA participants showed at least one significant pairwise comparison. For these participants, the range of effect sizes of surprisals change over time was 18.22 to 107.01, standard errors ranged from 2.52 to 33.70, and *p*-values ranged from < 0.0001 to 0.042.

**Figure 5:**
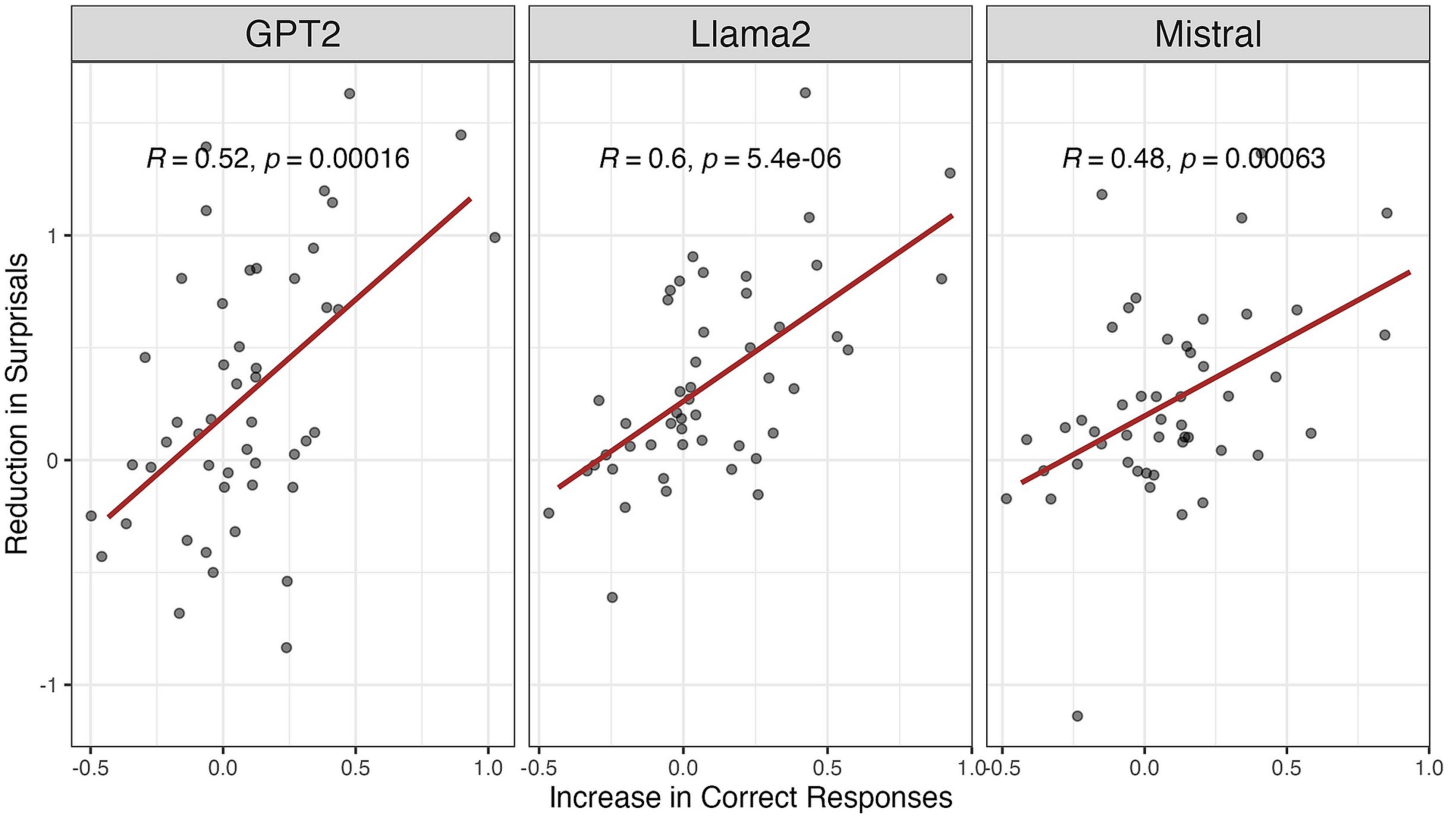
Correlations of the reduction in surprisals and the increase in correct responses scored by clinicians.

Figure 5 suggested that for PWA, greater reductions in surprisals are associated with increased accuracy in sentence production. This provides additional statistical evidence linking surprisals reduction to language recovery following structural priming training. Crucially, we found that this relationship generalizes to llama-2-7b and Mistral. In other words, PWA with more severe language impairments showed more pronounced improvements with structural priming training, as reflected in significant increases in accuracy, which were strongly positively correlated with reductions in surprisals. This inverse preference effect was consistently observed across multiple PLMs.

### 4.2 PLMs track syntax in language recovery

Study 2 results suggested that GPT-2 surprisals index beyond local, lexical level in language recovery, as shown by findings in error types and the relationships over time.

#### 4.2.1 PLM-indexed errors across time

Table 4 showed that the PWA group exhibited significant changes in surprisals from Pre-test to post-test sessions (coefficients of 0.926 and 0.964 with *p* < 0.001). AEM did not show significant changes in surprisals for their errors (GE, NS, and LE), likely because they generally made fewer errors regardless of time points. Recall that the majority of the “Incorrect” in AEM was grammatical, non-target PO, and only 3 out 564 “Incorrect” were ungrammatical errors.

**Table 4:** Surprisal of error productions across time.

Group	𝛽	*SE*	*p*
PWA			
Pre-test−Post-test 1	0.926	0.239	<0.0001
Pre-test−Post-test 2	0.964	0.245	<0.0001
Post-test 1−Post-test 2	0.039	0.213	0.982
AEM			
Pre-test−Post-test 1	−0.098	0.18	0.601

The insignificant decrease of surprisals in PWA productions with errors from Post-test 1 to Post-test 2 (Table 4) also reinforced the maintenance effect (c.f. Figure 4), suggesting that surprisals can effectively track language recovery in various stages. This also highlights the clinical interpretability of surprisals in characterizing production errors, further informing recovery trajectories.

#### 4.2.2 PLM-indexed errors across categories

Surprisals generated by GPT-2 showed differential correlations with various error types, revealing the categories of linguistic disruptions. GE (grammatical errors) showed the strongest positive correlation with surprisals (*r* = 0.277, *p* < 0.001), followed by non-sentential utterances missing a lexical verb (NS; *r* = 0.195, *p* < 0.001), suggesting that GPT-2 is particularly sensitive to structural violations that extend beyond isolated word-level anomalies. LE (lexical errors) showed a weaker but significant positive correlation (*r* = 0.088, *p* = 0.006), indicating that while lexical disruptions affect surprisal, their influence is less salient. No significant correlation was found between GPT-2 surprisals and NT_other (*r* = −0.025, *p* = 0.43), indicating that PLM-surprisals are not sensitive to other grammatical non-target structures.

Notably, when participants with aphasia produced more NT_po, surprisals tended to decrease, as indicated by the negative correlation. One likely reason is that we used conditional surprisals, which assumed the DO structure as the expected form. Despite their syntactic differences (NT_po and DO), NT_po and DO share a similar semantic meaning. Consequently, the surprisals of NT_po conditioned on DO should hypothetically be lower compared to those of other syntactically and semantically distinct productions. To investigate this further, we used the *sentence-transformers/all-MiniLM-L6-v2* model to embed sentence pairs into vector space representations (Sentence Transformers, 2024). Computational semantics studies have shown that such representations capture word content, and similarities between these representations capture content overlap (Yu and Ettinger, 2020). We thus calculated the cosine similarity between each target DO sentence and the corresponding participant production. The results showed that productions labeled as NT_po were highly similar to their DO targets (Mean = 0.902, SD = 0.116), indicating strong semantic overlap despite structural differences. In contrast, similarity scores were lower for other error types: GE (Mean = 0.782), LE (Mean = 0.738), and NS (Mean = 0.598). These results supported our hypothesis that the negative correlation between surprisals and NT_po frequency is possibly attributed to the fact that NT_po and DO differ syntactically but share semantic content. This also suggested that surprisals are sensitive to more than just semantics. It might serve as a composite score of syntactic and semantic information, with a stronger weighting toward syntax. If surprisals primarily tracked semantics, then error types such as GE and LE, would have shown similar correlation strengths. However, only GE exhibited a strong correlation, while LE did not, further indicating that PLM-surprisals are more responsive to syntactic divergence.

### 4.3 PLMs assess language recovery with few-shot prompting

**Figure 6:**
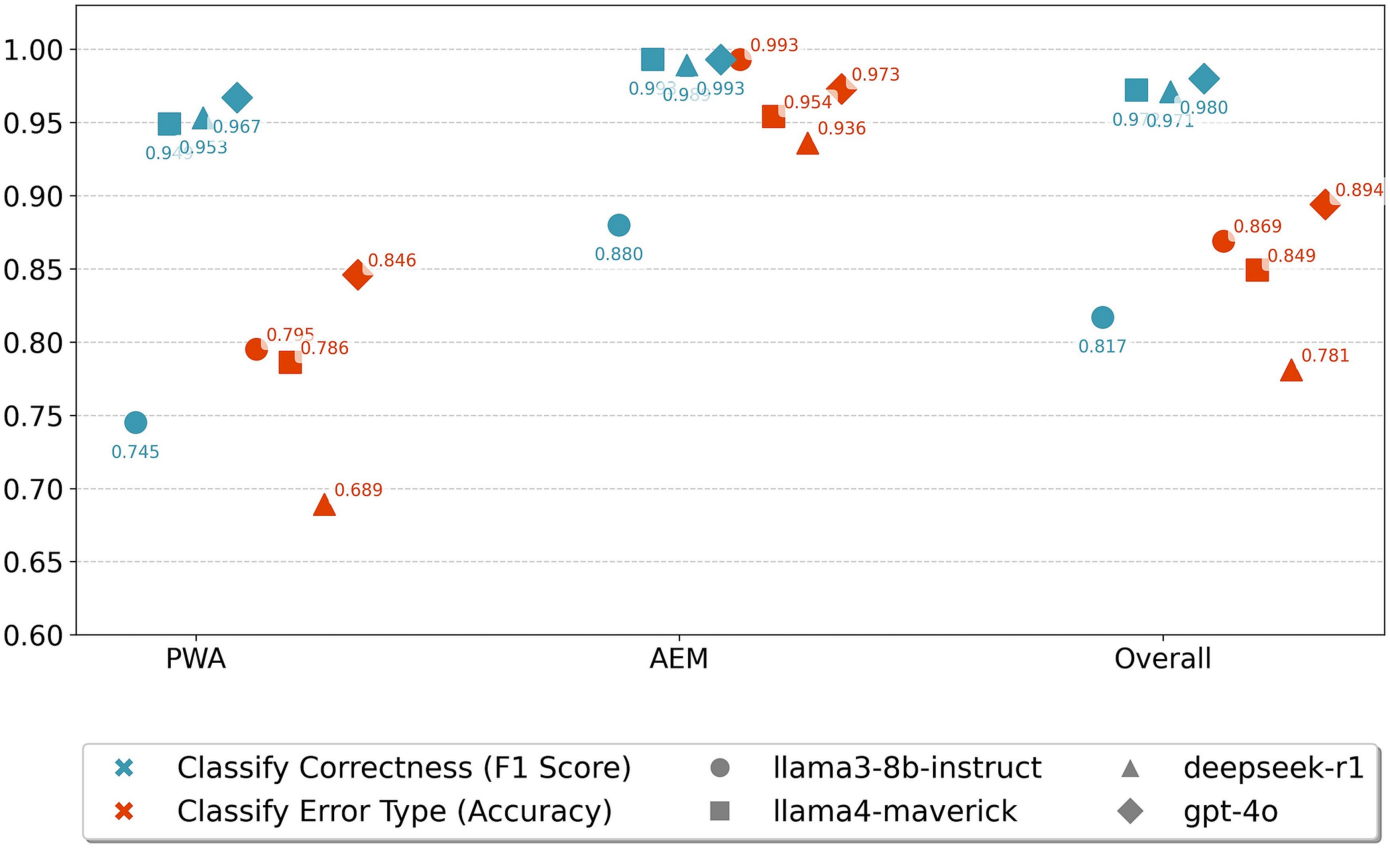
PLMs prompting performance in classifying sentence production correctness and error categories.

Figure 6 demonstrated that prompting PLMs to assess recovery-related sentence correctness and error categorization yields promising performance. Overall, our findings revealed distinct performance patterns across the evaluated PLMs, groups, and tasks.

First, regarding PLMs, most models achieved strong performance on correctness classification, with F1 scores generally exceeding 0.95, particularly llama4-maverick and gpt-4o which consistently performed above 0.97. However, llama3-8b demonstrated a unique performance profile that differed from all other models: while showing relatively lower correctness classification performance (F1 scores around 0.745–0.880), it exhibited comparatively better error type classification accuracy across conditions. This contrasting pattern suggested that llama3-8b may have developed different internal representations that capture more nuanced error-specific features, even when less decisive about binary correctness judgments.

Second, regarding groups, performance varied notably, with most PLMs showing superior results in the AEM group compared to PWA for both tasks. The PWA group proved more challenging, with several models showing performance drops, likely due to individual variance in PWA. For instance, deepseek-r1’s F1 score dropped to 0.689 for error classification in PWA, and llama3-8b achieved its lowest correctness F1 score (0.745) in PWA. We additionally reported the overall performance in Figure 6, where we combined both groups (AEM and PWA). Overall performance of PLMs on the whole dataset generally fell between AEM and PWA.

Third, the binary correctness classification task yielded a better performance than the multiclass error type classification across all PLMs for both AEM and PWA data. While correctness F1 scores consistently exceeded 0.90 for most PLM-group combinations, error type classification accuracy ranged from 0.689 to 0.894, highlighting the inherent complexity of teasing apart different error categories. This performance gap indicated that while PLMs can reliably detect whether language production contains errors, the fine-grained categorization of error types requires more specialized linguistic knowledge or domain-specific capabilities. Further, correctness is a binary classification task, whereas error-type classification requires distinguishing among multiple categories. As Jurafsky and Martin (2025) note, multiclass classification generally increases task difficulty, because models must learn and maintain more decision boundaries while coping with fewer training examples per class. In our case, this meant that PLMs had less data to represent each error type, making fine-grained classification more challenging than the binary detection of correctness.

## 5 Discussion

Most clinical applications of PLMs have focused on diagnostic classification, such as distinguishing individuals with language disorders from neuro-typical controls (Cong et al., 2024a,b). The present study takes a critical next step by exploring whether PLMs can be used not only for detection but also to track changes in language functioning over time. Shifting from binary diagnosis to continuous recovery monitoring is an important expansion of how PLMs can be leveraged in clinical contexts, particularly for capturing the dynamic and multidimensional nature of language rehabilitation.

### 5.1 PLMs track changes in sentence production: feasibility and efficacy

Our findings support our hypotheses that PLM-derived surprisals is both a feasible and effective index for tracking improvements in sentence production in aphasia. We conducted regression analyses alongside group-wise and time-point-wise comparisons, which revealed a marked reduction in conditional surprisals from Pre-test to both Post-test 1 and Post-test 2 sessions. This reduction was evident in both groups. Our results are consistent with previous findings on the efficacy of structural priming training, as observed in traditional clinical outcome measures such as production accuracy (Lee et al., 2019b, 2024; Mack et al., 2017) and syntactic preference choices (Cho-Reyes et al., 2016; Keen and Lee, 2022; Lee et al., 2019a). Importantly, PLM-surprisals were also sensitive to individuals’ learning trajectories relative to their Pre-test performance. At the group level, both PWA and AEM groups showed decreased surprisals following training, with the PWA group exhibiting a more pronounced effect. This finding is in line with previous studies showing increased priming-induced changes in their language production for speakers with less efficient linguistic knowledge (Branigan and Messenger, 2016; Cho-Reyes et al., 2016; van Boxtel et al., 2023; see also Hartsuiker and Kolk, 1998). That is, individuals with higher initial surprisal values, reflecting more deficits in sentence production before training, demonstrated greater improvement after training at both group and individual levels.

Moreover, across all three PLMs tested, reductions in surprisals were significantly correlated with training-induced improvements in sentence production. This correlation indicates the value and efficacy of PLM-surprisals in detecting individual differences in treatment response, supporting its potential use in tracking treatment effects in aphasia. Further, our individual-level analyses showed promising clinical implications for personalization of aphasia rehabilitation. Another advantage of using PLM-derived surprisals over traditional accuracy-based or categorical error-type analyses lies in its capacity to provide a continuous, graded measure of recovery. This continuous scale allows for the detection of subtle improvements or shifts in linguistic complexity that binary metrics may miss. As such, surprisals might be a more sensitive and fine-grained index of language rehabilitation than binary accuracy stores.

Lastly, our prompting-based approach extends prior work incorporating probability and instruction-based prompting for PLM interaction (Hu and Levy, 2023). Our findings also resonate with the prompting study by Imaezue and Marampelly (2025), which proposed a simulation method to improve conversational agents by incorporating clinically relevant speech error patterns, thereby aligning AI-driven dialogues more closely with the needs of aphasia. Prompting introduces an end-to-end pipeline: users provide simple instruction prompts with few-shot examples, and the model generates output labels, either numerical or categorical, without requiring intermediate annotations. This streamlined interface may significantly enhance the clinical applicability of PLMs. Our results demonstrate that prompting PLMs is both a feasible approach for language rehabilitation in medical settings. While our findings are based on four specific PLMs, we hypothesize they would generalize to stronger models, provided they are autoregressive and trained on a word prediction objective.

Hybrid approaches could further improve prompting’s efficacy. Our findings demonstrated robust performance in binary correctness classification, but with a relatively lower accuracy rate in error-type classification. We maintain that a hybrid approach, integrating probabilistic metrics (such as surprisals) with prompting, could augment the precision of fine-grained error detection. For instance, employing probability as intermediate outputs to guide PLMs in determining the statistical range and distribution of each distinct error type, and subsequently utilizing these probabilities by PLMs for enhanced error type classification.

### 5.2 PLMs track different error types: reliability and interpretability

Our findings suggest the reliability and interpretability of PLM-surprisals, which are crucial in medical contexts. Error categorization allowed us to pinpoint what PLMs surprisals reliably capture, leading to meaningful results that both clinicians and computational linguists can interpret. As discussed in previous studies, structural priming not only facilitates the immediate use of primed sentence structures but also induces lasting changes in language production in aphasia. Our study extended these insights by demonstrating that higher PLM-derived surprisals are associated with increased grammatical errors and non-sentential utterances, which are often seen in patients with syntactic deficits. These findings resonate with prior work (Rezaii et al., 2019, 2022a, 2022b, 2023; Cong et al., 2024a,b), which identified computational linguistic indices can serve as interpretable measures of the structural deficits underlying aphasia.

Cong et al. (2024a) showed that PLM-surprisals index both word- and sentence-level disruptions characteristic of agrammatic aphasia, such as reduced mean length of utterance, lower syntactic complexity (e.g., fewer embedded clauses), and diminished verb usage. These features were more strongly associated with PLM-surprisals than with classic clinical measures such as lexical diversity. Rezaii et al. (2023) suggested that high surprisal values may also reflect a communication strategy, where PWA, constrained by limited processing resources, rely more heavily on high-frequency content words and simplified syntax. These are strategies that may go unnoticed in categorical error analyses. Our current findings expand Cong et al. (2024a) and support the proposal that PLM-surprisals function as an integrative metric that reflects broader deficits in syntactic planning and structural composition, not merely lexical retrieval. Taken together, PLM-surprisals offer a reliable and interpretable utility for quantifying language recovery beyond linear, surface-level correctness, enabling a hierarchical, finer-grained characterization of structural impairments and recovery trajectories in aphasia.

Surprisals reduction could be interpreted as lexical repetition or memorization, rather than alignment with the intended syntactic target. In our study, we attempted to address this by using conditional surprisal measures that presupposed the DO structure, and by conducting additional analyses with embedding representations (Yu and Ettinger, 2020; sentence transformer 2024; as in Section 4.2.2). Our findings suggested that surprisals were more responsive to structural alignment and syntactic divergence than to (lexical) overlap and repetition. Future work could control for such effects more directly, for example, by systematically varying lexical items across test points, or by incorporating analyses that leverage (semantically or syntactically) nonce words, which explicitly separate lexical from structural contributions.

Further, our analyses revealed that, at the group level, PWA exhibited significant changes in surprisals associated with improvements in syntactic production, while AEM participants maintained lower error rates overall. Crucially, within the PWA group, the correlations between specific error types (e.g., grammatical errors versus non-target productions) and surprisal values revealed that as participants’ productions became more target-like, overall surprisals decreased. This observation indicates that the PLM-surprisal measure is sensitive not only to the accuracy of syntactic reproduction but also to the reduction in error load, which is a key indicator of language recovery.

### 5.3 Limitations and future work

This study has some limitations that point toward future directions. Our sample included only participants with mild-to-moderate aphasia, leaving open the question of whether surprisals-based measures remain valid in more severe cases. Severe aphasia often involves a higher proportion of non-sentential or unintelligible utterances, which may challenge both PLM-surprisals and prompting-based classification by reducing linguistic structure and reliable input for probability estimation. Adaptations such as more robust preprocessing, error-tolerant parsing, or training models on data representative of severely impaired speech will likely be necessary, and validation in severe populations remains critical. Beyond sentence-level structures, our methods could be extended to syntactic forms commonly studied in priming research, such as passives, relative clauses, locative alternations, and noun phrase modifiers (e.g., Cleland and Pickering, 2003; Chang et al., 2012; van Gompel et al., 2012; Mahowald et al., 2016), as well as to discourse production, which has not yet been systematically characterized with PLMs. Future work could evaluate whether these approaches generalize across syntactic and communicative contexts, thereby broadening understanding of priming-based treatment effects.

Our current design also did not include a control group of people with aphasia who did not undergo priming training. Prior studies have established priming’s role in healthy participants (e.g., Branigan and McLean, 2016; Savage et al., 2006; Heyselaar and Segaert, 2022; Bock and Griffin, 2000; Chang et al., 2012), but future research might want to directly isolate priming’s effects in clinical contexts by contrasting PWA with and without training. Additionally, careful attention is needed to speech variability, multimodality of data, differences across PLM pre-training paradigms, and comorbidities (e.g., neurological versus. Psychiatric illness), all of which may influence surprisals measures.

We focused on few-shot prompting, guided by empirical evidence and recommendations in natural language processing. Few-shot examples were selected by domain experts (aphasia researchers and annotators), and prompt templates were adapted for two tasks: detecting correctness and detecting error types. We did not explore alternative prompting strategies (e.g., chain-of-thought, tree-of-thought), as our aim was to examine the clinical efficacy of PLMs with simple few-shot prompting. Similarly, we chose not to disclose whether data came from controls or PWA. We maintained that labels could simplify the task and that lengthy prompts describing group characteristics might risk distracting PLMs. Instead, we prompted PLMs on the combined dataset and analyzed performance by group and overall (Section 4.3; Figure 6). We acknowledge that future research could benefit from broader comparisons across prompting strategies, the inclusion of labeled datasets, and systematic variation in prompt design. Such work would extend our findings and provide a more thorough understanding of prompting choices in aphasia treatment assessments.

At the same time, recent advances in AI offer a concrete path toward deployment in speech-language therapy. Interactive systems such as ChatGPT have been used to facilitate word retrieval (Purohit et al., 2023), while Aphasia-GPT demonstrated integration of generative AI into augmentative and alternative communication (AAC) to enhance accessibility and personalization (Bailey et al., 2024). Building on this trajectory, our findings show how PLM-derived surprisals and prompting-based pipelines can provide interpretable, scalable tools for monitoring clinical progress (e.g., Hou et al., 2025). By quantifying surprisal as a syntactic and lexical metric, evaluating model performance with intrinsic (e.g., surprisals shift, error detection) and extrinsic (e.g., F1 scores, accuracy) metrics, and automating error classification, our approach demonstrates a feasible pathway for reducing the burden of clinical evaluations.

For real-world adoption, several steps might be critical. Clinician training through workshops and modular sessions will be essential to build familiarity with interpreting automated indices. User-centered dashboards should present outputs as intuitive visualizations and summaries, integrated with therapy software and remote platforms, while supporting multimodal input (Bailey et al., 2024; Imaezue and Marampelly, 2025). Embedding these tools into EHR systems would allow structured tracking of longitudinal outcomes, ensure interoperability, and consolidate treatment notes and indices in one place (Wang et al., 2019; Gale et al., 2024; Cohen et al., 2020). Such integration would enable data-driven, scalable, and accessible rehabilitation monitoring while maintaining explainability in high-risk domains.

Taken together, while further validation is required across severity levels, syntactic contexts, and clinical designs, the integration of PLM-surprisals and prompting pipelines represents a promising step toward precision rehabilitation. With careful attention to clinical variability, user-centered implementation, and responsible deployment, this approach has the potential to improve accessibility and quality of aphasia therapy worldwide.

## 6 Conclusion

Our study demonstrated PLM as an effective and interpretable utility for tracking language recovery in aphasia with gradient sensitivity. Monitoring PLMs over time can provide a quantifiable measure of treatment efficacy, which is particularly useful for tailoring interventions to individual patients. Overall, the convergence of computational systems with clinical measurement suggests the potential of PLM-based approaches to refine our understanding of language recovery.

## Data Availability

The original contributions presented in the study are included in the article/Supplementary material, further inquiries can be directed to the corresponding author.
