# Supplementary material for: Tracking priming-induced language recovery in aphasia with pre-trained language models
Source: Front Artif Intell. 2025 Oct 30;8:1668399. doi: 10.3389/frai.2025.1668399 (PMC12611953; doi:10.3389/frai.2025.1668399)
Supplement: Supplementary file 2 [file Presentation_1.ZIP › Prompting_demo.pdf]

Prompting LLMs

few-shot learning with Llama series

Correctness

Evaluate the Target Production against the Target Sentence by following the step-by-step instructions. instruction: Return 1 if the candidate uses a correct Double Object (DO) structure that preserves the target's meaning; otherwise return 0.

steps: [
{'step': 1, 'description': "If the sentence explicitly uses a preposition like 'to' or 'for' to link the Theme and the Recipient/Goal, return 0."},
{'step': 2, 'description': 'Verify that the sentence uses Double Object (DO) structure: Agent, Verb, Recipient/Goal, Theme (Noun-Verb-Noun-Noun).', 'details': ['Slight word order variations (e.g., adverbs, auxiliaries) are acceptable if core argument roles are preserved.', 'Modifiers (e.g., adjectives, determiners) may vary as long as the Recipient and Theme roles are intact.'], 'action': 'If the structure clearly deviates from DO or inserts a prepositional phrase, return 0.'},
{'step': 3, 'description': 'Check meaning preservation', 'criteria': ['Articles, tense, and auxiliary verb differences are acceptable.', 'Ignore typos and allow synonyms or paraphrases listed below.', 'The same semantic roles must be assigned to the correct participants (Agent, Recipient, Theme).'], 'action': 'If meaning is preserved despite variations, return 1; otherwise, return 0.'}]

allowed_synonyms: {'nouns': {'man': ['guy', 'boy', 'young man'], 'woman': ['lady', 'girl'], 'thief': ['robber'], 'athlete': ['sport guy'], 'dancer': ['ballerina'], 'singer': ['performer'], 'queen': ['princess'], 'wife': ['woman'], 'husband': ['man'], 'policeman': ['cop', 'police officer'], 'mailman': ['postman'], 'girl': ['child (if no other child in the picture)'], 'bishop': ['priest'], 'waitress': ['server', 'waiter'], 'cheerleader': ['girl']}, 'verbs': {'transitive': {'weigh': ['measure'], 'push': ['shove'], 'lift': ['pick up'], 'touch': ['pat']}, 'dative': {'give': ['hand', 'offer', 'reward'], 'award': ['give', 'hand'], 'offer': ['serve'], 'feed': ['serve']}}} examples: [{'target': 'The man is giving the woman the cake.', 'candidate': 'The man gave the woman the cake.', 'expected_output': 1}, {'target': 'The man is giving the woman the cake.', 'candidate': 'The woman is giving the man the cake.', 'expected_output': 0}, {'target': 'The man is giving the woman the cake.', 'candidate': 'The man gave a woman a cake.', 'expected_output': 1}, {'target': 'The man is giving the woman the cake.', 'candidate': 'The man gives the cake to the woman.', 'expected_output': 0}, {'target': 'The woman is making the man the cake.', 'candidate': 'The woman made the cake for the woman.', 'expected_output': 0}]

Now, evaluate the following:
Target Sentence: The daughter offers the man the coffee. Target Production: The daughter offered coffee to the man

Reasoning steps

To evaluate the Target Production against the Target Sentence, we will follow the given step-by-step instructions.
1. **Step 1:** Check if the sentence explicitly uses a preposition like 'to' or 'for' to link the Theme and the Recipient/Goal. - The Target Production is: "The daughter offered coffee to the man." - It uses the preposition 'to', so according to Step 1, we should return 0.
2. Since we have already returned 0 in Step 1, there is no need to proceed to Step 2 and Step 3.

Final output

Therefore, based on the instructions provided, the evaluation of the Target Production "The daughter offered coffee to the man" against the Target Sentence "The daughter offers the man the coffee" is **0**.

ErrorType

Label each candidate sentence based on its structure and meaning in relation to the target sentence. Follow the steps below in order. Assign only one label: NT_po, NT_other, GE, LE, NS, or Other. note: PO means Prepositional Object; DO means Double Object. Apply steps sequentially; return on first match.

step: 1 condition: Grammatical PO (Agent-Verb-Theme + preposition 'at/to/for/in' + Recipient/Goal) label: NT_po example: {'target': 'He gave her the cake.', 'candidate': 'He gave the cake to her.'}
step: 2 condition: Grammatical, neither DO nor PO label: NT_other example: {'target': 'He sent her a message.', 'candidate': 'She got a message from him.'}
step: 3 condition: Ungrammatical PO (missing or wrong prepositions, arguments, word order) or DO with morpho-syntactic errors label: GE examples: [{'target': 'He gave the fireman the bucket.', 'candidate': 'He gave the fireman.'}, {'target': 'He gave the mayor a speech.', 'candidate': 'He gave a speech to the mayor.'}, {'target': 'She sent him an email.', 'candidate': 'She sent an email.'}, {'target': 'The lifeguard fed the fairy the spaghetti.', 'candidate': 'The lifeguard is feeding the fairy to the spaghetti.'}]
step: 4 condition: DO order with wrong noun or verb altering meaning label: LE example: {'target': 'He gave the girl a cookie.', 'candidate': 'He is giving pirate the cookie.'}
step: 5 condition: Not a full sentence (no verb or fragment) label: NS example: {'target': 'The clown gave the girl a balloon.', 'candidate': 'Clown girl.'}
step: 6 condition: None apply or multiple errors combined label: Other

Now, evaluate the following:
Target Sentence: The daughter offers the man the coffee. Target Production: The daughter offered coffee to the man

Reasoning steps

To evaluate the given target sentence and its production, let's follow the steps provided. 1. **Target Sentence:** "The daughter offers the man the coffee." 2. **Target Production:** "The daughter offered coffee to the man"
First, we need to analyze the structure and meaning of both sentences.
- The target sentence is in the present tense and follows a Double Object (DO) structure: "The daughter offers [the man] [the coffee]."
- The target production is in the past tense and follows a Prepositional Object (PO) structure: "The daughter offered [coffee] to [the man]." Let's apply the steps:
Step 1: Check if the candidate (Target Production) is a grammatical PO structure.
- The target production "The daughter offered coffee to the man" is grammatically correct and follows the PO structure (Agent-Verb-Theme + preposition 'to' + Recipient). The change from present to past tense does not affect the PO structure check directly, as the condition primarily concerns the grammatical structure and the presence of a preposition.

Final output

Thus, it matches the NT_po condition because it is a grammatical PO structure related to the target sentence's DO structure.

Label: NT_po Since it matches the first condition, we do not need to proceed further. The label for the given target production "The daughter offered coffee to the man" in relation to the target sentence "The daughter offers the man the coffee" is **NT_po**.
